# Estudo da Reatividade Microvascular em Pacientes Hipertensos com Adiposidade Corporal Elevada

**DOI:** 10.36660/abc.20190364

**Published:** 2020-11-01

**Authors:** Jenifer d'El-Rei, Michelle Rabello Cunha, Samanta de Souza Mattos, Bianca Cristina Marques, Viviane Prangiel de Menezes, Ana Rosa Cunha, Érica Monteiro França, Wille Oigman, Mario Fritsch Neves

**Affiliations:** 1 Universidade do Estado do Rio de Janeiro Clínica Médica Rio de JaneiroRJ Brasil Universidade do Estado do Rio de Janeiro - Clínica Médica, Rio de Janeiro, RJ - Brasil

**Keywords:** Hipertensão/efeitos do tratamento, Adiposidade, Endotélio, Permeabilidade Capilar

## Abstract

**Fundamento:**

Diversos índices antropométricos têm sido propostos para determinar a associação entre excesso de peso e fatores de risco cardiovascular.

**Objetivo:**

Avaliar a relação entre adiposidade corporal e reatividade microvascular em pacientes hipertensos sob terapia anti-hipertensiva.

**Métodos:**

Pacientes hipertensos tratados de 40 a 70 anos foram submetidos à avaliação de índices antropométricos: conicidade (IC), adiposidade corporal (IAC), adiposidade visceral (IAV) e relação cintura-estatura (RCE). Os participantes foram divididos pelos tercis de percentual de gordura (%G) obtido pela bioimpedância elétrica (BIA) e submetidos a teste de reatividade microvascular (*laser speckle contrast image*), medida da velocidade da onda de pulso (VOP). O valor de p < 0,05 foi considerado estatisticamente significativo.

**Resultados:**

A variação da área sob a curva (ASC) da perfusão cutânea foi inferior no tercil superior (97 ± 57% *vs.* 67 ± 36%; p = 0,027). O %G apresentou correlação significativa com RCE (r = 0,77; p < 0,001), IAV (r = 0,41; p = 0,018), IC (r = 0,60; p < 0,001) e IAC (r = 0,65; p < 0,001) nos homens e somente com RCE (r = 0,55; p < 0,001) e IAC (r = 0,60; p < 0,001) nas mulheres. Na regressão linear, a ASC mostrou associação independente com o %G (β =–3,15; p = 0,04) nas mulheres e com a glicemia (β = –1,15; p = 0,02) nos homens. Não houve diferença nas medidas de VOP.

**Conclusão:**

Os índices antropométricos de obesidade foram mais associados ao %G nos homens. A maior adiposidade corporal foi relacionada com menor reatividade microvascular, o que foi mais evidente nas mulheres. Não houve diferença na rigidez arterial, o que pode ter sido influenciado pelo tratamento anti-hipertensivo.

## Introdução

A Organização Mundial da Saúde (OMS) aponta a obesidade como um dos maiores problemas de saúde pública no mundo e, no Brasil, ela vem crescendo cada vez mais. Evidências apontam que mais de 50% da população apresentam-se na faixa de sobrepeso e obesidade.^1^^,^^2^

O diagnóstico correto de obesidade ou sobrepeso requer algumas formas de quantificação da composição corporal. As técnicas de imagem são alternativas que oferecem maior precisão; no entanto, a simplicidade de utilização destaca os métodos antropométricos como bons instrumentos para avaliação da gordura corporal. Diante disso, diversos índices antropométricos têm sido propostos para determinar a associação entre excesso de peso e fatores de risco cardiovascular.^3^

O índice de massa corporal (IMC) é o que tem maior divulgação em estudos, mas não se correlaciona à distribuição da gordura corporal. Assim, as medidas da circunferência da cintura (CC) e a razão cintura-quadril (RCQ) são os indicadores mais utilizados na aferição da distribuição central do tecido adiposo. Outros que vêm demonstrando forte ligação com os fatores de risco cardiovascular são: o conicidade (IC), a relação cintura-estatura (RCE) e, mais recentemente, a adiposidade corporal (IAC) e o adiposidade visceral (IAV).^3^

Na obesidade, o tecido adiposo perivascular torna-se altamente inflamado e induz disfunção vascular por secreção aumentada de fatores de vasoconstrição, como os principais componentes do sistema renina-angiotensina e adipocinas pró-inflamatórias, os quais são importantes contribuintes para ativação endotelial e inflamação vascular.^4^

Na prática clínica, é essencial a identificação de parâmetros que possam refletir, de maneira mais precisa e viável, a distribuição do tecido adiposo (visceral ou subcutâneo) e sua relação com as alterações metabólicas e inflamatórias que levam ao prejuízo da saúde vascular e, consequentemente, aumentam o risco cardiovascular. Desse modo, este estudo teve como objetivo avaliar a relação entre adiposidade corporal e reatividade microvascular em pacientes hipertensos sob terapia anti-hipertensiva, além de correlacionar o percentual de gordura corporal e a influência do sexo aos índices antropométricos de adiposidade e risco cardiovascular.

## Métodos

### População do estudo

Pacientes hipertensos com idade entre 40 e 70 anos, de ambos os sexos, em uso de anti-hipertensivo por pelo menos quatro semanas, foram selecionados e admitidos em um estudo transversal. Os critérios de exclusão foram IMC ≥ 40 kg/m², diabetes melito, terapia de reposição hormonal e uso de betabloqueador ou estatina. Para análise dos resultados, os pacientes foram divididos de acordo com o tercil do %G, diferenciado pelo sexo. Nas mulheres, o ponto de corte dos tercis foi de 36,49 e 39,87%, enquanto nos homens, foi de 25,27 e 28,95%. O protocolo foi aprovado pelo Comitê de Ética local (50751314.9.0000.5259), e todos os participantes leram e assinaram o termo de consentimento livre e esclarecido em conformidade com o descrito na resolução 466/2012.

### Avaliação nutricional

Seguindo as técnicas preconizadas pela OMS, o peso corporal foi aferido em balança digital da marca Filizola^®^ com capacidade máxima de 180 kg.^5^ Na mesma balança foi verificada a estatura, a partir do antropômetro. O IMC foi calculado dividindo-se o peso corporal (em quilograma) pelo quadrado da altura (Alt; em metro). Os pontos de corte adotados para a classificação nutricional foram baseados nos critérios propostos pela OMS.^6^

As circunferências da cintura e do quadril foram obtidas com auxílio de fita métrica inextensível. A CC foi determinada no ponto médio entre a última costela e a crista ilíaca. A avaliação da circunferência do quadril (CQ) foi realizada no maior diâmetro da região glútea. A partir dessas medidas, foi calculada a RCQ.^6^

O cálculo da RCE foi feito conforme fórmula RCE = CC/Alt, e o cálculo do IC foi realizado utilizando a fórmula^7^ IC = CC/0,109 x √ Peso/Alt.

O IAC foi calculado a partir da medida da CQ e da estatura:^8^ IAC (%G) = CQ/(Alt x √Alt) – 18.

O IAV foi calculado levando em consideração as variações por gênero:^9^

- Homens: IAV = (CC/39,68 + 1,88 x IMC) x (TG/1,03) x (1,31/HDL)
- Mulheres: IAV = (CC/39,68 + 1,89 x IMC) x (TG/0,81) x (1,52/HDL).

Em que TG = triglicerídeo (mmol/l); HDL = lipoproteina de alta densidade (do inglês, *high density lipoprotein*) (mmol/l).

A BIA foi realizada com o aparelho tetrapolar Biodynamics^®^ modelo 310e, utilizado para avaliação do %G, seguindo recomendações prévias.^10^

### Avaliação laboratorial

Amostras de sangue venoso foram coletadas após jejum de 8 horas. Glicose sérica, creatinina, colesterol total, HDL e TG foram medidos com uma técnica de autoanálise (Technicon DAX96, Miles Inc). A proteína C reativa (PCR) foi aferida pelo método de turbidimetria. A avaliação da função renal foi realizada utilizando a taxa de filtração glomerular (TFG), estimada pela equação *Chronic Kidney Disease-Epidemiology Collaboration* (CKD-EPI).^11^ A insulina foi medida por radioimunoensaio, e o índice de *homeostatic model assessment-insulin resistance* (HOMA-IR) = [glicemia de jejum (mmol/l) x insulina de jejum (mUI/ml)/22,5] foi usado para estimar a sensibilidade à insulina.^12^

### Avaliação de pressão arterial e idades vascular e cardiometabólica

As medidas da pressão arterial sistólica (PAS) e diastólica (PAD) foram obtidas com aparelho digital calibrado (modelo HEM-705CP, OMRON Healthcare Inc., Illinois), realizadas com o paciente em posição sentada e após cinco minutos de repouso. O cálculo de idade vascular foi baseado no *Framingham Heart Study*.^13^

A idade cardiometabólica foi obtida pelo endereço eletrônico https://myhealthcheckup.com, acessando o item *Cardiometabolic Age*.^14^ A síndrome metabólica foi definida de acordo com os critérios estabelecidos pelo Programa Nacional de Educação e Tratamento sobre Colesterol (NCEP ATP III).^15^

### Reatividade microvascular

A reatividade microvascular foi avaliada utilizando o método *laser speckle contrast image* (LSCI) (Pericam PSI System, Perimed, Suécia), em combinação com a hiperemia reativa pós-oclusão (HRPO) para análise contínua das alterações de perfusão cutânea microvasculares dependentes de endotélio expressas em unidades de perfusão arbitrárias (UPA). Com essas análises, foram obtidos a média do período basal, a ASC do período basal de um minuto, a média do pico de HRPO e a ASC do período de um minuto após a oclusão. A condutância vascular cutânea (CVC) foi obtida pela fórmula: CVC = perfusão basal (ou HRPO)/pressão arterial média (PAM).

### Parâmetros hemodinâmicos centrais

A análise da onda de pulso da artéria radial foi feita utilizando um dispositivo de tonometria disponível comercialmente (SphygmoCor; AtCor Medical, Sydney, Austrália). O aumento de pressão (AP) é a diferença entre a segunda e a primeira pressão de pico sistólico, e o *augmentation index* (Aix) é definido como a razão entre o AP e a pressão de pulso aórtica.

### Velocidade da onda de pulso

As ondas de pulso foram obtidas transcutaneamente pelo aparelho COMPLIOR-SP (Alam Medical, France), por meio de transdutores colocados sobre a carótida direita e, ao mesmo tempo, sobre a artéria femoral direita. A distância entre os pulsos carotídeo e femoral foi medida diretamente com o auxílio de uma fita métrica inextensível, sendo o valor multiplicado por 0,8 para fins de cálculo da VOP. Essa medida foi corrigida calculando a VOP normalizada (VOP-CF-N), com a seguinte fórmula: VOP-CF-N = (VOP–CF/PAM) x 100.^16^

### Análise estatística

Os resultados foram expressos em média ± desvio-padrão. Para determinação do tamanho da amostra para este estudo, foi considerada a equivalência da variação da dilatação mediada por fluxo (DMF) observada em obesos. Desse modo, para uma diferença de 3,0% na DMF, desvio-padrão de 4,0%, com 80% de poder de estudo e significância em 5%, um número mínimo de 22 participantes em cada grupo seria necessário. Considerando uma perda estimada de 10% da amostra, a quantidade mínima foi definido em 72 participantes. O teste de *Shapiro-Wilk* foi utilizado para avaliar distribuição normal. Os tercis do %G foram comparados pelo teste *One-Way ANOVA*, seguido pelo pós-teste de *Tukey*. As variáveis categóricas foram apresentadas como frequência e percentual e comparadas pelo teste do Qui-quadrado. O coeficiente de *Pearson* foi obtido em cada teste de correlação entre as variáveis contínuas. Foi considerado um intervalo de confiança de 95%, sendo estatisticamente significativo quando p < 0,05. A regressão linear foi realizada respeitando os pressupostos necessários, incluindo a ausência de multicolinearidade, considerando a ASC como variável dependente, ajustada por idade e por PAS, e realizada separadamente nos grupos de homens e mulheres. As análises estatísticas foram feitas pelo programa *Statistical Package for the Social Sciences* (SPSS) versão 20.0 para *Windows* (SPSS, Chicago, IL).

## Resultados

Os resultados apresentados a seguir referem-se aos 81 pacientes que foram incluídos no estudo, com média de idade de 58 ± 6 anos, sendo 59% do sexo feminino (n = 48). A média do risco cardiovascular foi de 16,8 ± 11,2%, e a da pressão arterial, 138 ± 11/83 ± 9 mmHg. Os parâmetros clínicos da população dividida por tercis são encontrados na Tabela 1. Não houve diferença significativa na média de idade e risco cardiovascular entre os grupos. As idades vascular e cardiometabólica foram significativamente maiores no último tercil quando comparado ao primeiro.

**Tabela 1:** Parâmetros clínicos divididos pelos tercis do percentual de gordura

Parâmetros	Percentual de gordura			Valor de p
	1º tercil (n = 27)	2º tercil (n = 27)	3º tercil (n = 27)	
Idade (anos)	57 ± 6	58 ± 7	60 ± 7	0,116
RCV (%)	15,6 ± 10,5	14,2 ± 9,9	20,8 ± 12,9	0,079
Idade vascular (anos)	70 ±11	68 ± 12	77 ± 10* ††	0,007
Idade CM (anos)	55 ± 7	55 ± 8	60 ± 8*	0,025
PAS (mmHg)	136 ± 9	135 ± 13	140 ± 11	0,173
PAD (mmHg)	84 ± 8	81 ± 10	86 ± 8	0,137
Pressão de pulso (mmHg)	52 ± 6	54 ± 9	54 ± 8	0,608
**Estilo de vida, n (%)**				
Etilismo	11 (41)	12 (44)	13 (48)	0,861
Sedentarismo	22 (82)	19 (70)	22 (82)	0,526
**Uso de anti-hipertensivo, n (%)**				
Diurético	26 (96)	25 (93)	26 (96)	0,769
ISRA	23 (85)	25 (93)	24 (89)	0,687
ACC	8 (30)	5 (19)	5 (19)	0,526
Monoterapia	4 (15)	2 (7)	4 (15)	0,493
Com dois fármacos	16 (59)	22 (82)	18 (67)	
Com três fármacos	7 (26)	3 (11)	5 (19)	

Dwwados expressos em média ± desvio-padrão ou em proporções, quando indicado. Valor de p corresponde ao Qui-quadrado para variáveis categóricas e One-Way Anova para variáveis numéricas com pós-teste de Tukey, sendo

* p < 0,05 vs. 1º tercil,

†† p < 0,01 vs. 2º tercil. RCV: risco cardiovascular; CM: cardiometabólica; PAS: pressão arterial sistólica; PAD: pressão arterial diastólica; ISRA: inibidor do sistema renina-angiotensina; ACC: antagonista do canal de cálcio.

O IMC foi bem maior no terceiro tercil, em relação ao primeiro e segundo. A CC foi maior no segundo e terceiro tercis, comparada à do primeiro, no sexo masculino, e no terceiro em relação ao primeiro no sexo feminino. A RCQ foi significativamente maior nas mulheres do terceiro tercil em relação ao primeiro, não sendo encontradas diferenças entre os homens. A RCE foi maior no último tercil quando comparado aos demais, tanto nos homens quanto nas mulheres.

O IC foi maior no último tercil no sexo masculino, quando comparado ao primeiro, e no último no sexo feminino quando comparado ao segundo e primeiro tercis. O IAC foi significativamente maior no terceiro tercil quando comparado aos outros dois. Já o IAV foi maior no terceiro tercil quando comparado ao segundo. O número de critérios para classificação da síndrome metabólica foi significativamente maior no último tercil em relação ao segundo (Tabela 2).

**Tabela 2:** Parâmetros de adiposidade corporal divididos pelo tercil do percentual de gordura

Parâmetros	Percentual de gordura			Valor de p
	1º tercil (n = 27)	2º tercil (n = 27)	3º tercil (n = 27)	
IMC (kg/m^2)^	26,1 ± 3,7	28,9 ± 3,1	31,4 ± 2,8*** †	< 0,001
**Perímetro cintura (cm)**				
♂	88,9 ± 11,7	98,8 ± 6,6*	106,3 ± 8,5***	< 0,001
♀	86,5 ± 8,3	91,5 ± 6,2	97,7 ± 8,8***	< 0,001
**RCQ**				
♂	0,88 ± 0,08	0,93 ± 0,05	0,95 ± 0,04	0,053
♀	0,80 ± 0,05	0,82 ± 0,05	0,86 ± 0,06*	0,040
RCE				
♂	0,53 ± 0,07	0,56 ± 0,03	0,62 ± 0,05** †	0,002
♀	0,54 ± 0,05	0,57 ± 0,04	0,63 ± 0,05*** ††	< 0,001
**Gordura corporal (%)**				
♂	20,0 ± 4,6	26,7 ± 1,1***	31,8 ± 2,5*** ††	< 0,001
♀	31,0 ± 4,5	38,5 ± 1,0**	44,6 ± 8,4*** ††	< 0,001
**Índice de conicidade**				
♂	1,25 ± 0,87	1,30 ± 0,57	1,33 ± 0,62*	0,026
♀	1,21 ± 0,76	1,21 ± 0,53	1,28 ± 0,80* †	0,009
**Índice de adiposidade corporal**				
♂	28,0 ± 3,7	27,4 ± 1,4	31,9 ± 3,7* ††	0,004
♀	34,3 ± 3,4	36,4 ± 3,2	40,9 ± 4,6*** ††	< 0,001
Índice de adiposidade visceral	2,88 ± 1,13	2,51 ± 1,04	3,55 ± 1,98†	0,037
Critérios SM	2,3 ± 1,1	2,1 ± 0,9	2,9 ± 0,9†	0,018

Dados expressos em média ± desvio-padrão. Valor de p corresponde ao Qui-quadrado para variáveis categóricas e One-Way Anova para variáveis numéricas com pós-teste de Tukey, sendo

* p < 0,05;

** p < 0,01;

*** p < 0,001 vs. 1º tercil;

† p < 0,05;

†† p < 0,01 vs. 2º tercil.

IMC: índice de massa corporal; RCQ: razão cintura-quadril; RCE: relação cintura-estatura; SM: síndrome metabólica.

♂ sexo masculino;

♀ sexo feminino.

A Tabela 3 apresenta os dados laboratoriais sem diferença significativa em creatinina, perfis lipídico e glicêmico, PCR e TFG entre os grupos. O ácido úrico e a relação TG/HDL foram significativamente maiores no último tercil.

**Tabela 3:** Parâmetros laboratoriais divididos pelo tercil do percentual de gordura

Parâmetros	Percentual de gordura			Valor de p
	1º tercil (n = 27)	2º tercil (n = 27)	3º tercil (n = 27)	
Creatinina (mg/dl)	0,88 ± 0,20	0,89 ± 0,20	0,92 ± 0,25	0,851
Ácido úrico (mg/dl)	5,29 ± 1,60	5,52 ± 1,49	6,40 ± 1,69*	0,029
Colesterol total (mg/dl)	209 ± 47	203 ± 28	216 ± 36	0,485
HDL (mg/dl)	56 ± 16	61 ± 20	51 ± 19	0,164
LDL (mg/dl)	126 ±38	123 ± 43	129 ± 34	0,822
TG (mg/dl)	131 ± 49	111 ± 47	130 ± 58	0,061
TG/HDL	2,60 ± 1,43	2,28 ± 1,92	3,59 ± 2,85†	0,050
Glicose (mg/dl)	94 ± 11	93 ± 10	96 ± 11	0,670
Insulina (mcU/ml)	14,4 ± 7,2	13,8 ± 4,8	16,5 ± 7,4	0,317
HOMA-IR	3,38 ± 1,69	3,20 ± 1,29	3,91 ± 1,89	0,270
PCR-us (mg/dl)	0,71 ± 0,50	0,73 ± 0,49	0,83 ± 0,59	0,655
TFG (ml/min/1,73 m²)	87 ± 13	84 ± 19	79 ± 19	0,544

Dados expressos em média ± desvio-padrão. Valor de P corresponde ao Qui-quadrado para variáveis categóricas e One-Way Anova para variáveis numéricas com pós-teste de Tukey, sendo

* p < 0,05 vs. 1º tercil;

† p < 0,05 vs. 2º tercil.

HDL: lipoproteína de alta densidade; LDL, lipoproteína de baixa densidade; TG, triglicerídeo; HOMA-IR, homeostatic model assessment-insulin resistance; PCR-us, proteína C reativa ultrassensível; TFG, taxa de filtração glomerular.

Na Tabela 4 encontram-se os dados dos testes vasculares. Nos parâmetros hemodinâmicos centrais, não foram encontradas diferenças significativas entre os grupos. A VOP-CF e a VOP-CF-N não apresentaram diferenças estatísticas entre os tercis. Os dados obtidos pelo LSCI não demonstraram diferenças entre os grupos em perfusão, ASC e CVC basal e na HRPO. A variação da ASC foi significativamente menor no terceiro tercil quando comparado ao primeiro.

**Tabela 4:** Parâmetros vasculares divididos pelo tercil do percentual de gordura

Parâmetros	Percentual de gordura			
	1º tercil (n = 27)	2º tercil (n = 27)	3º tercil (n = 27)	Valor de p
	**Hemodinâmica central Tonometria de aplanação**			
PAS aórtica (mmHg)	131 ± 15	130 ± 17	132 ± 16	0,855
PP aórtica (mmHg)	46 ± 11	47 ± 10	46 ± 12	0,941
AP (mmHg)	17 ± 10	16 ± 7	15 ± 7	0,746
Aix (%)	33 ± 11	32± 10	32 ± 10	0,945
Aix @ FC 75 (%)	27 ± 9	27 ± 9	27 ± 9	0,997
DE (%)	34 ± 4	35 ± 3	35 ± 4	0,723
SEVR	167 ± 37	156 ± 27	155 ± 26	0,272
	**VOP**			
VOP-CF (m/s)	10,0 ± 1,8	9,7 ± 1,9	9,7 ± 2,0	0,803
VOP-CF-N (m/s)	9,9 ± 1,7	9,8 ± 1,7	9,4 ± 1,9	0,468
	**LSCI**			
Perfusão basal (UAP)	28,4 ± 11,0	32,2 ± 10,7	30,9 ± 9,8	0,410
Basal CVC (UPA/mmHg)	0,28 ± 0,11	0,33 ± 0,12	0,30 ± 0,09	0,303
Perfusão HRPO (UPA)	84,2 ± 26,2	87,2 ± 22,2	90,6 ± 21,0	0,601
HRPO CVC (UPA/mmHg)	0,84 ± 0,28	0,89 ± 0,25	0,87 ± 0,20	0,743
ASC basal (UPA)	1800 ± 679	2001 ± 663	1996 ± 579	0,334
ASC HRPO (UPA)	3360 ± 1190	3257 ± 856	3261 ± 882	0,978
Variação de ASC (%)	97 ± 57	70 ± 35	67 ± 36*	0,027
Variação de CVC (%)	218 ± 105	185 ± 73	211 ± 90	0,366

Dados expressos em média ± desvio-padrão. Valor de p corresponde ao Qui-quadrado para variáveis categóricas e One-Way Anova para variáveis numéricas com pós-teste de Tukey, sendo

* p < 0,05 vs. 1º tercil.

PAS: pressão arterial sistólica; PP: pressão de pulso; AP: aumento de pressão; Aix: augmentation index; DE: duração de ejeção; SEVR: razão de viabilidade subendocárdica; VOP: velocidade da onda de pulso; CF: carótida femoral; CF-N: carótida femoral normalizada; LSIC: laser speckle de imagem contrastada; UPA: unidade de perfusão arbitrária; CVC: condutância vascular cutânea; HRPO: hiperemia reativa pós-oclusão; ASC: área sob a curva.

**Figura 1:**
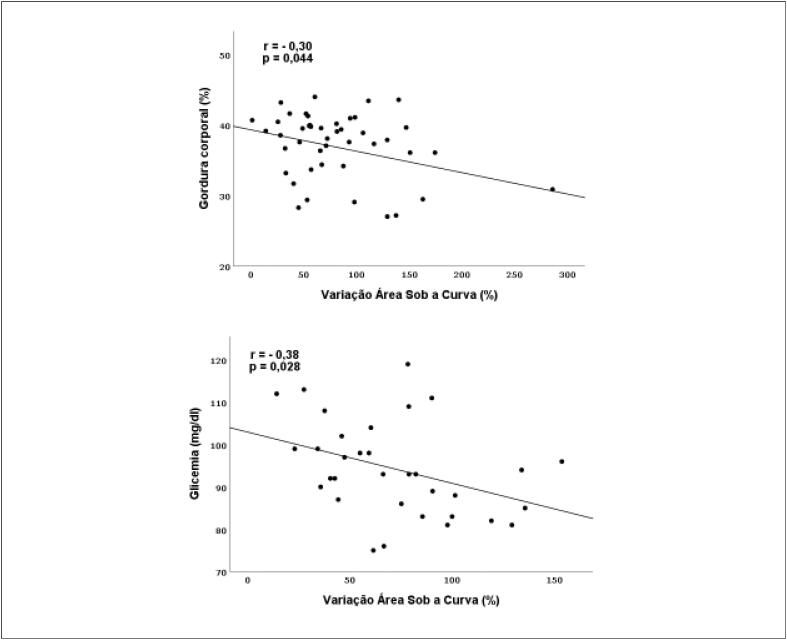
Correlação da variação da área sob a curva da perfusão cutânea pelo método laser speckle contrast image com gordura corporal em mulheres e com glicemia em homens.

O %G exibiu correlações positivas e significativas com IMC, CC, RCE e índice de adiposidade, tanto nas mulheres quanto nos homens. Além desses resultados, entre os homens, a RCQ, o IC, o IAV e o número de critérios para síndrome metabólica também apresentaram correlação positiva e significativa com o %G corporal (Tabela 5). A variação da ASC apresentou correlação negativa e significativa com o %G corporal nas mulheres e com a glicemia entre os homens (Figura 1). Na regressão linear, essas associações permaneceram independentes, mesmo após ajustes para idade e PAS (Tabela 6).

**Tabela 5:** Correlação de Pearson dos índices de adiposidade e de risco cardiovascular com o percentual de gordura, por sexo

Variáveis	Mulheres (n = 48)		Homens (n = 33)	
	Coeficiente r	Valor de p	Coeficiente r	Valor de p
IMC (kg/m²)	0,556	< 0,001	0,738	< 0,001
CC (cm)	0,476	0,001	0,767	< 0,001
RCQ	0,215	0,152	0,505	0,003
RCE	0,550	< 0,001	0,767	< 0,001
Índice de adiposidade corporal	0,599	< 0,001	0,653	< 0,001
Índice de conicidade	0,264	0,076	0,597	< 0,001
Índice de adiposidade visceral	−0,037	0,809	0,410	0,018
IV Framingham (anos)	0,062	0,682	−0,005	0,976
Idade CM (anos)	0,242	0,109	−0,044	0,810
Critérios SM	−0,066	0,662	0,464	0,007

Correlação de Pearson. IMC: índice de massa corporal; CC: circunferência da cintura; RCQ: razão cintura-quadril; RCE: relação cintura-estatura; IV: idade vascular; CM: cardiometabólica; SM: síndrome metabólica.

**Tabela 6:** Regressão linear da variável dependente da variação da área sob a curva da perfusão cutânea pelo método laser speckle contrast image com gordura corporal no sexo feminino e com glicemia no sexo masculino, após ajuste para idade e pressão arterial sistólica.

Variáveis dependentes		Variáveis independentes	Coeficiente não padronizado B	IC 95%	Coeficiente padronizado Beta	Valor de p
ASC (%)	♀	Gordura corporal (%)	−3,15	−6,29-0,10	−0,32	0,049
	♂	Glicemia (mg/dl)	−1,35	−2,47-0,22	−0,43	0,020

ASC: área sob a curva;

♂ sexo masculino;

♀ sexo feminino.

## Discussão

Este estudo avaliou a relação da adiposidade corporal com a reatividade microvascular e suas associações com diferentes índices antropométricos e metabólicos em uma população de hipertensos sob terapia medicamentosa. No que diz respeito aos parâmetros pressóricos, tanto periféricos quanto centrais, não foram encontradas diferenças entre os grupos, o que mostra que, do ponto de vista hemodinâmico, eles estavam bem equilibrados.

As idades vascular e cardiometabólica foram maiores no tercil superior de %G, evidência de uma associação positiva entre acúmulo de gordura corporal e danos vasculares e metabólicos. Considerando que não houve diferença significativa no cálculo do risco cardiovascular, esse achado reforça a importância da estimativa desses parâmetros.

Valores superiores de ácido úrico e da relação TG/HDL foram encontrados no tercil de maior %G, quando comparado com os tercis inferiores. A elevação do ácido úrico tem sido associada à síndrome metabólica. Estudos experimentais sugeriram que o ácido úrico pode penetrar nas fibras musculares lisas e vasculares, culminando no aumento da expressão de mediadores inflamatórios. As consequências são elevação da pressão arterial e hipertrofia de células musculares lisas vasculares.^17^^–^^19^ Um estudo publicado recentemente, realizado na Índia com indivíduos adultos, demonstrou associação dos níveis de ácido úrico com parâmetros antropométricos de obesidade, como IMC, RCQ e RCE.^18^

A relação TG/HDL tem sido proposta como um simples marcador de resistência à insulina, funcionando como um biomarcador para identificar perfis de risco cardiometabólico.^20^ Pantoja-Torres et al.^21^ demonstraram, em uma população adulta eutrófica, associação positiva da relação TG/HDL com resistência insulínica. Essa relação também foi estudada por Baez-Duarte et al.^22^ mas em uma população adulta com IMC médio de 27,8 Kg/m², na qual foi observada associação entre a relação TG/HDL e a menor sensibilidade à insulina e a presença de síndrome metabólica.

Uma vez que a disfunção endotelial é considerada um marcador do processo aterosclerótico, é crucial avaliar suas manifestações mais precoces em micro e macrocirculação.^23^ A avaliação da função endotelial pela reatividade microvascular, por meio do método LSCI, não vem sendo utilizada nos ensaios clínicos com população obesa.

A reatividade microvascular na amostra estudada foi associada de modo negativo ao acúmulo de tecido adiposo, e a variação da ASC foi 30% inferior no terceiro tercil, quando comparada à do primeiro. Suboc et al.^24^ demonstraram que a obesidade foi relacionada com pior função endotelial em adultos não diabéticos. Esses achados só se tornaram significativos quando os grupos foram divididos por sexo. A função endotelial, avaliada neste estudo pela variação da ASC, correlacionou-se à glicemia no sexo masculino, sugerindo uma possível associação importante entre função endotelial e sensibilidade à insulina nos homens. Já nas mulheres, essa correlação mostrou-se mais evidente com o %G corporal, indicando uma provável relação direta entre tecido adiposo e função vascular no sexo feminino. Essas correlações se mantiveram após ajustes para a idade e para PAS, importantes fatores na regulação da função endotelial.

Com relação aos parâmetros de rigidez arterial, não foram encontradas diferenças entre os grupos estudados. Desamericq et al.^25^ não encontraram relação entre obesidade e aumento de rigidez arterial em uma amostra de adultos com fatores de risco cardiovascular associados, como diabetes tipo 2. Menezes et al.^26^ também não observaram associação entre obesidade e resistência insulínica com alteração vascular, tanto na função endotelial quanto na rigidez arterial. No estudo, a ligação da adiposidade com a rigidez arterial pode ter sido atenuada pela administração de fármacos vasoprotetores no esquema terapêutico dos pacientes, como os inibidores do sistema renina-angiotensina (ISRA).

O %G obtido pela BIA no sexo masculino mostrou uma boa relação com todos os índices antropométricos de avaliação de adiposidade corporal e com o número de critérios para síndrome metabólica. Nas mulheres, tal correlação permaneceu somente para IMC, CC, RCE e IAC. Em 2007, um estudo realizado no Brasil, com o objetivo de determinar a relação entre os diversos indicadores de obesidade e o risco coronariano, mostrou que os indicadores apresentaram forte associação com RCE, destacando-se entre os homens o RCQ e o IC, enquanto para as mulheres entre 50 e 74 anos o IC foi o melhor marcador. Essa diferença pode ser explicada pela menopausa, fase em que ocorre perda da proteção fornecida pelo hormônio estrogênio, levando ao maior acúmulo de gordura abdominal, que contribui para a ocorrência de problemas cardiovasculares.^27^

Algumas limitações foram identificadas no estudo. A ausência de marcadores inflamatórios e adipocinas prejudicou a análise da inflamação como mecanismo da disfunção endotelial associada à adiposidade corporal aumentada. A PCR foi o único marcador avaliado e não se mostrou diferente entre os grupos estudados. Os efeitos de alguns anti-hipertensivos podem ter influenciado esse resultado. Nenhum método de imagem foi utilizado para quantificar o tecido adiposo visceral. Entretanto, o objetivo principal do trabalho foi utilizar métodos mais simples de avaliação da adiposidade corporal e sua associação com as alterações vasculares que podem caracterizar maior risco cardiovascular.

## Conclusão

Na amostra apresentada de pacientes hipertensos em tratamento, os índices antropométricos de obesidade foram mais associados ao %G corporal entre os homens. O maior risco cardiovascular entre aqueles com maior adiposidade corporal foi mais evidenciado pelas maiores idades vascular e cardiometabólica nesse grupo de pacientes. A maior adiposidade corporal foi relacionada com menor reatividade microvascular, o que foi mais evidente entre as mulheres. Não houve diferença em relação à rigidez arterial, o que pode ser atribuído ao uso das medicações anti-hipertensivas que mantiveram níveis semelhantes de pressão arterial nos grupos estudados.
