# Supplementary material for: Estudo da Reatividade Microvascular em Pacientes Hipertensos com Adiposidade Corporal Elevada
Source: Arq Bras Cardiol. 2020 Nov 1;115(5):896–904. [Article in Portuguese] doi: 10.36660/abc.20190364 (PMC8452213; doi:10.36660/abc.20190364)
Supplement: Translation

# Translation: Microvascular Reactivity in Hypertensive Patients with High Body Adiposity

**Authors:** Jenifer d'El-Rei, Michelle Rabello Cunha, Samanta de Souza Mattos, Bianca Cristina Marques, Viviane Prangiel de Menezes, Ana Rosa Cunha, Érica Monteiro França, Wille Oigman, Mario Fritsch Neves

**Affiliations:** 1 Rio de JaneiroRJ Brazil Universidade do Estado do Rio de Janeiro - Clínica Médica, Rio de Janeiro, RJ - Brazil

**Keywords:** Hypertension/drug effects, Adiposity, Endothelium, Capillary Permeability

## Abstract

**Background:**

Several anthropometric indexes have been proposed to determine the association between overweight and cardiovascular risk factors.

**Objective:**

To evaluate the relationship between body adiposity and microvascular reactivity in hypertensive patients under antihypertensive therapy.

**Methods:**

Treated hypertensive patients aged 40 to 70 were submitted to evaluation of anthropometric indexes: conicity (CI), body adiposity (BAI), visceral adiposity (VAI) and waist-to-height ratio (WHtR). Participants were divided by the terciles of fat percentage (%F) obtained by bioelectrical impedance. The patients underwent microvascular reactivity test (Laser Speckle Contrast Image) and pulse wave velocity (PWV) measurement. The p value <0.05 was considered statistically significant.

**Results:**

The variation of the area under the curve (AUC) of the skin perfusion was lower in the upper tercile (97±57% vs. 67±36%; p=0.027). %F showed significant correlation with WHtR (r=0.77; p<0.001), VAI (r=0.41; p=0.018), CI (r=0.60; p<0.001), BAI (r=0.65; p<0.001) in men and only with WHtR (r=0.55; p<0.001) and BAI (r=0.60; p<0.001) in women. In linear regression, AUC was independently associated with %F (β=−3.15; p=0.04) in women and with blood glucose (β=−1.15; p=0.02) in men. There was no difference in PWV measurements.

**Conclusion:**

Anthropometric indices were more associated with %F in men. Higher body adiposity was associated with lower microvascular reactivity, which was more evident in women. There was no difference in arterial stiffness, which may have been influenced by antihypertensive treatment.

## Introduction

The World Health Organization (WHO) considers obesity a major public health problem worldwide. In Brazil, obesity is growing increasingly, and epidemiological evidence shows that more than 50% of the population is overweight and obese.^1^^,^^2^

Correct diagnosis of obesity or overweight requires some ways of quantifying body composition. Imaging techniques are alternatives that offer greater precision in the assessment of fat accumulation, but the simplicity of use emphasizes anthropometric methods as good tools for body fat assessment. Several anthropometric indices have been proposed to determine the association between overweight and cardiovascular risk factors.^3^

Body mass index (BMI) is the most widely reported indicator in studies, but it does not correlate with body fat distribution. Waist circumference (WC) and waist-hip ratio (WHR) measurements are the most commonly used markers for central distribution of adipose tissue. Other indicators that have been showing strong correlation with cardiovascular risk factors are: conicity index (CI), waist-to-height ratio (WHtR) and, more recently, body adiposity (BAI) and visceral (VAI) indices.^3^

In obesity, perivascular adipose tissue promotes inflammation and induces vascular dysfunction through increased secretion of vasoconstriction factors, such as the main components of the renin-angiotensin system and proinflammatory adipokines, which are important contributors to endothelial activation and vascular inflammation.^4^

In clinical practice, it is essential to identify parameters that may reflect the distribution of adipose tissue (visceral or subcutaneous) more accurately and feasibly, and its relationship with metabolic and inflammatory changes that lead to impaired vascular health and, consequently, increase cardiovascular risk. Thus, this study aimed to evaluate the relationship between body adiposity and microvascular reactivity in hypertensive patients under antihypertensive therapy, and to correlate body fat percentage and sex influence on anthropometric indices of adiposity and cardiovascular risk.

## Methods

### Study Population

Hypertensive patients aged 40 to 70, of both sexes, using antihypertensive drugs for at least four weeks, were selected from our outpatient clinic and admitted to a cross-sectional study. Exclusion criteria were BMI ≥40 kg/m², diabetes mellitus, hormone replacement therapy, and betablocker or statin use. For analysis of the results, the patients were divided according to terciles of body fat percentage, differentiated by gender. In females, the terciles cutoff points were 36.49 and 39.87%, while in males they were 25.27 and 28.95%. The protocol was approved by the local Ethics Committee (50751314.9.0000.5259), and all participants read and signed the informed consent in accordance with Declaration 466/2012.

### Nutritional Assessment

Body weight was measured on a Filizola^@^ digital scale with maximum capacity of 180 Kg, following the techniques recommended by the WHO.^5^ In the same scale, height was verified by the anthropometer. BMI was calculated by dividing body weight (in kilograms) by the square of height (Ht; in meters). The cutoff points adopted for the nutritional classification were based on the criteria proposed by the WHO.^6^

Waist and hip circumferences were obtained with the aid of an inextensible tape measure. Waist circumference was determined at the midpoint between the last rib and the iliac crest. Hip circumference (HC) assessment was performed on the largest diameter of the gluteal region. After obtaining these measurements, the WHR was calculated.^6^

The WHtR was calculated according to the following formula: WHtR=WC/Ht

The CI calculation was performed using the following formula:^7^

Cl=WC/0.109 x√Weight/Ht.

The BAI was calculated from the measurement of hip circumference and height:^8^ BAI (%fat)=HC/(Ht×√Ht)−18

The VAI was calculated considering the variations by gender:^9^

$$\begin{array}{l} \begin{array}{l} \text{Men: VAI}=(\text{WC}/39.68+1.88\times \text{BMI})\times (\text{TG}/1.03)\times \\ \left(1.31/\text{HDL}\right) \end{array}\\ \begin{array}{l} \text{Women: VAI}=(\text{WC}/39.68+1.89\times \text{BMI})\times (\text{TG}/0.81)\\ \times (1.52/\text{HDL}) \end{array} \end{array}$$

Where: TG = Triglyceride (mmol/l); HDL = High-density lipoprotein (mmol/l).

Bioelectrical impedance analysis (BIA) was performed with the Biodynamics model 310e tetrapolar device and was used to evaluate body fat percentage, following previous recommendations.^10^

### Laboratory tests

Venous blood samples were collected after an 8-hour fast. Serum glucose, creatinine, total cholesterol, HDL and TG were measured with a self-analysis technique (Technicon DAX96, Miles Inc). C-reactive protein (CRP) was measured by the turbidimetry method. Renal function was assessed using glomerular filtration rate (GFR) estimated by the Chronic Kidney Disease-Epidemiology Collaboration (CKD-EPI) equation.^11^ Insulin was measured by radioimmunoassay, and the Homeostatic Model Assessment Index — Insulin Resistance (HOMA-IR) = [fasting glucose (mmol/L) x fasting insulin (mUI/mL)/22.5] was used to estimate insulin sensitivity.^12^

### Blood pressure assessment, vascular and cardiometabolic ages

Systolic (SBP) and diastolic (DBP) blood pressure measurements were obtained with a calibrated digital device (model HEM-705CP, OMRON Healthcare Inc., Illinois), performed with the patient in a sitting position and after five minutes of rest. Vascular age was based on the Framingham Heart Study.^13^

Cardiometabolic age was obtained from https://myhealthcheckup.com, accessing Cardiometabolic Age.^14^ Metabolic syndrome was defined according to the criteria established by the National Cholesterol Education and Treatment Program (NCEP ATP III).^15^

### Microvascular Reactivity

Microvascular reactivity was assessed using the Laser Speckle Contrast Image (LSCI) method (Pericam PSI System, Perimed, Sweden) in combination with Post Occlusive Reactive Hyperemia (PORH) for continuous analysis of expressed endothelium-dependent microvascular skin perfusion changes in arbitrary perfusion units (APU). Through these analyzes, we obtained the mean perfusion and area under the curve (AUC) at 1-min baseline period, PORH peak mean, and the area under the curve at 1-min after occlusion. Cutaneous vascular conductance (CVC) was obtained by dividing baseline perfusion (or PORH) by mean arterial pressure (MAP).

### Central Hemodynamic Parameters

Radial artery pulse wave analysis was performed using a commercially available tonometry device (SphygmoCor; AtCor Medical, Sydney, Australia). Augmentation pressure (AP) is the difference between the second and first systolic peak pressure, and augmentation index (Aix) is defined as the ratio between AP and aortic pulse pressure.

### Pulse Wave Velocity (PWV)

Pulse waves were obtained transcutaneously by the Complior Analyses device (Alam Medical, France) using transducers placed over the right carotid artery and the right femoral artery at the same time. The distance between the carotid and femoral pulses was measured directly with an inextensible tape measure, which was multiplied by 0.8 to calculate the carotid-femoral PWV. This measurement was corrected by calculating normalized carotid-femoral PWV (PWV-N) using the following formula: PWV-N=(PWV/MAP) x 100.^16^

### Statistical Analysis

Results were expressed as mean ± standard deviation. To determine the sample size of this study, we considered the equivalence of the change in Flow Mediated Dilatation (FMD) observed in obese subjects. Thus, for a difference of 3.0 (%) in FMD, a standard deviation of 4.0 (%), with 80% study power and 5% significance, a minimum of 22 participants in each group would be required. Considering an estimated loss of 10% of the sample, the minimum number was set at 72 participants. Shapiro-Wilk test was used to evaluate normal distribution. The terciles of fat percentage were compared by One-Way ANOVA test, followed by Tukey post-test. Categorical variables were presented as frequency and percentage, and compared using the chi-square test. Pearson's coefficient was obtained in each correlation test between continuous variables. A confidence interval of 95% was considered, being statistically significant when p<0.05. Linear regression was performed respecting the necessary assumptions, including the absence of multicollinearity, considering the UAC as a dependent variable, adjusted for age and SBP, and performed separately in the groups of men and women. Statistical analyses were performed using the Statistical Package for Social Sciences (SPSS) version 20.0 for Windows (SPSS, Chicago, IL).

## Results

The results presented below are related to 81 patients included in this study, with a mean age of 58±6 years, 59% female (n=48). The average cardiovascular risk (CVR) was 16.8±11.2% and mean blood pressure was 138±11/83±9 mmHg. Clinical parameters of the groups divided by terciles are shown in Table 1. There was no significant difference in mean age and CVR among the groups. Vascular and cardiometabolic ages were significantly higher in the last tercile compared to the first.

**Table 1:** Clinical parameters in the groups divided by terciles of fat percentage

	Fat percentage			
Parameters	1^st^ Tercile (n=27)	2^nd^ Tercile (n=27)	3^rd^ Tercile (n=27)	p value
Age (years)	57 ± 6	58 ± 7	60 ± 7	0.116
FRS (%)	15.6 ± 10.5	14.2 ± 9.9	20.8 ± 12.9	0.079
Vascular age (years)	70 ±11	68 ± 12	77 ± 10* ††	0.007
CM age (years)	55 ± 7	55 ± 8	60 ± 8*	0.025
Systolic BP (mmHg)	136 ± 9	135 ± 13	140 ± 11	0.173
Diastolic BP (mmHg)	84 ± 8	81 ± 10	86 ± 8	0.137
Pulse pressure (mmHg)	52 ± 6	54 ± 9	54 ± 8	0.608
**Lifestyle, n (%)**				
Alcohol use No physical activity	11 (41) 22 (82)	12 (44) 19 (70)	13 (48) 22 (82)	0.861 0.526
**Antihypertensive use, n (%)**				
Diuretics	26 (96)	25 (93)	26 (96)	0.769
RASI	23 (85)	25 (93)	24 (89)	0.687
CCA	8 (30)	5 (19)	5 (19)	0.526
Monotherapy	4 (15)	2 (7)	4 (15)	0.493
With 2 drugs	16 (59)	22 (82)	18 (67)	
With 3 drugs	7 (26)	3 (11)	5 (19)	

Data expressed as mean ± standard deviation or in proportions where indicated. P value corresponds to chi-square for categorical variables and One-Way Anova for numeric variables with Tukey's post-test,

* p <0.05 vs. 1^st^ tercile,

†† p <0.01 vs. 2^nd^ tercile.

FRS: Framingham risk score; CM: cardiometabolic; BP: blood pressure; RASI: renin-angiotensin system inhibitor; CCA,: calcium channel antagonist.

The BMI was significantly higher in the third tercile compared to the first and second. WC was significantly higher in the second and third terciles compared to the first in males, and in the third compared to the first in females. The WHR was significantly higher in the third tercile than in the first in women, and no differences were found between men. The WHtR was significantly higher in the last tercile compared to the others in both men and women.

The CI was higher in the last when compared to the first tercile in males, and in the last compared to the second and first terciles in females. The BAI was significantly higher in the third tercile compared to the other two groups.

The VAI was significantly higher in the third compared to the second tercile. The number of criteria for metabolic syndrome was significantly higher in the last compared to the second tercile (Table 2).

**Table 2:** Body adiposity indexes divided by fat percentage terciles

	Fat percentage			
Parameters	1^st^ Tercile (n=27)	2^nd^ Tercile (n=27)	3^rd^ Tercile (n=27)	p value
BMI (kg/m^2^)	26.1 ± 3.7	28.9 ± 3.1	31.4 ± 2.8*** †	< 0.001
**Waist circumference (cm):**				
♂	88.9 ± 11.7	98.8 ± 6.6*	106.3 ± 8.5***	< 0.001
♀	86.5 ± 8.3	91.5 ± 6.2	97.7 ± 8.8***	< 0.001
**WHR:**				
♂	0.88 ± 0.08	0.93 ± 0.05	0.95 ± 0.04	0.053
♀	0.80 ± 0.05	0.82 ± 0.05	0.86 ± 0.06*	0.040
**WHtR:**				
♂	0.53 ± 0.07	0.56 ± 0.03	0.62 ± 0.05** †	0.002
♀	0.54 ± 0.05	0.57 ± 0.04	0.63 ± 0.05*** ††	< 0.001
**Body fat (%):**				
♂	20.0 ± 4.6	26.7 ± 1.1***	31.8 ± 2.5*** ††	< 0.001
♀	31.0 ± 4.5	38.5 ± 1.0**	44.6 ± 8.4*** ††	< 0.001
**Conicity index:**				
♂	1.25 ± 0.87	1.30 ± 0.57	1.33 ± 0.62*	0.026
♀	1.21 ± 0.76	1.21 ± 0.53	1.28 ± 0.80* †	0.009
**Body adiposity index:**				
♂	28.0 ± 3.7	27.4 ± 1.4	31.9 ± 3.7* ††	0.004
♀	34.3 ± 3.4	36.4 ± 3.2	40.9 ± 4.6*** ††	< 0.001
Visceral adiposity index	2.88 ± 1.13	2.51 ± 1.04	3.55 ± 1.98†	0.037
MS criteria	2.3 ± 1.1	2.1 ± 0.9	2.9 ± 0.9†	0.018

Note: Data expressed as mean ± standard deviation. P value corresponds to chi-square for categorical variables and One-Way Anova for numeric variables with Tukey's post-test, where

* p <0.05,

** p <0.01,

*** p <0.001 vs. 1^st^ tercile;

† p <0.05,

†† p <0.01 vs. 2^nd^ tercile.

BMI: body mass index; WHR: waist to hip ratio; WHtR: waist to height ratio; MS: metabolic syndrome.

♂ male;

♀ female.

Table 3 presents the laboratory data with no significant difference in creatinine, lipid and glycemic profile, CRP and GFR among the groups. Uric acid and TG/HDL ratio were significantly higher in the last tercile group.

**Table 3:** Biochemical parameters divided by fat percentage terciles

	Fat percentage			
Parameters	1^st^ Tercile (n=27)	2^nd^ Tercile (n=27)	3^rd^ Tercile (n=27)	p value
Creatinine (mg/dl)	0.88 ± 0.20	0.89 ± 0.20	0.92 ± 0.25	0.851
Uric acid (mg/dl)	5.29 ± 1.60	5.52 ± 1.49	6.40 ± 1.69*	0.029
Total cholesterol (mg/dl)	209 ± 47	203 ± 28	216 ± 36	0.485
HDL-cholesterol (mg/dl)	56 ± 16	61 ± 20	51 ± 19	0.164
LDL-cholesterol (mg/dl)	126 ±38	123 ± 43	129 ± 34	0.822
TG (mg/dl)	131 ± 49	111 ± 47	130 ± 58	0.061
TG/HDL	2.60 ± 1.43	2.28 ± 1.92	3.59 ± 2.85†	0.050
Glucose (mg/dl)	94 ± 11	93 ± 10	96 ± 11	0.670
Insulin (mcU/ml)	14.4 ± 7.2	13.8 ± 4.8	16.5 ± 7.4	0.317
HOMA-IR	3.38 ± 1.69	3.20 ± 1.29	3.91 ± 1.89	0.270
us-CRP (mg/dl)	0.71 ± 0.50	0.73 ± 0.49	0.83 ± 0.59	0.655
eGFR (ml/min/1.73 m²)	87 ± 13	84 ± 19	79 ± 19	0.544

Data expressed as mean ± standard deviation. P value corresponds to chi-square for categorical variables and One-Way Anova for numeric variables with Tukey's post-test, where

* p<0.05 vs. 1^st^ tercile;

† p<0.05 vs. 2^nd^ tercile.

HDL: high-density lipoprotein; LDL: low-density lipoprotein; TG: triglyceride; HOMA-IR: Homeostatic Model Assessment — Insulin Resistance; us-CRP: ultrasensitive C-reactive protein; eGFR: estimated glomerular filtration rate.

Table 4 shows the vascular test results. Concerning the central hemodynamic parameters, no significant differences were found between the groups. PWV and PWV-N showed no statistical differences among the groups of terciles. Data obtained by LSCI showed no difference among the groups in baseline and PORH perfusion AUC and CVC. The variation in AUC was significantly lower in the third compared to the first tercile.

**Table 4:** Vascular parameters divided by fat percentage terciles

Parameters	Fat percentage			
	1^st^ Tercile (n=27)	2^nd^ Tercile (n=27)	3^rd^ Tercile (n=27)	p value
	**Central hemodynamics Applanation tonometry**			
Aortic SP (mmHg)	131 ± 15	130 ± 17	132 ± 16	0,855
Aortic PP (mmHg)	46 ± 11	47 ± 10	46 ± 12	0,941
AP (mmHg)	17 ± 10	16 ± 7	15 ± 7	0,746
Aix (%)	33 ± 11	32± 10	32 ± 10	0,945
Aix @ HR75 (%)	27 ± 9	27 ± 9	27 ± 9	0,997
ED (%)	34 ± 4	35 ± 3	35 ± 4	0,723
SEVR	167 ± 37	156 ± 27	155 ± 26	0,272
	**VOP**			
PWV (m/s)	10,0 ± 1,8	9,7 ± 1,9	9,7 ± 2,0	0,803
PWV-N (m/s)	9,9 ± 1,7	9,8 ± 1,7	9,4 ± 1,9	0,468
	**LSCI**			
Baseline perfusion (APU)	28,4 ± 11,0	32,2 ± 10,7	30,9 ± 9,8	0,410
Baseline CVC (APU/mmHg)	0,28 ± 0,11	0,33 ± 0,12	0,30 ± 0,09	0,303
PORH perfusion (APU)	84,2 ± 26,2	87,2 ± 22,2	90,6 ± 21,0	0,601
PORH CVC (APU/mmHg)	0,84 ± 0,28	0,89 ± 0,25	0,87 ± 0,20	0,743
Baseline AUC (APU)	1800 ± 679	2001 ± 663	1996 ± 579	0,334
PORH AUC (APU)	3360 ± 1190	3257 ± 856	3261 ± 882	0,978
AUC variation (%)	97 ± 57	70 ± 35	67 ± 36*	0,027
CVC variation (%)	218 ± 105	185 ± 73	211 ± 90	0,366

Data expressed as mean ± standard deviation. P value corresponds to One-Way Anova with Tukey's post-test, where

* p <0.05 vs. 1st tercile.

SP: systolic pressure; PP: pulse pressure; AP: augmentation pressure; Aix: augmentation index; ED: ejection duration; SEVR: subendocardial viability ratio; PWV: pulse wave velocity; PWV-N: normalized pulse wave velocity; LSCI: laser speckle contrast image; APU: arbitrary perfusion unit; CVC: cutaneous vascular conductance; PORH: post-occlusive reactive hyperemia; AUC: area under the curve.

Fat percentage showed positive and significant correlations with BMI, WC, WHtR and adiposity index in both women and men. In addition to these results, WHR, CI, VAI, and the number of criteria for metabolic syndrome also showed a positive and significant correlation with body fat percentage in men (Table 5). The variation in AUC showed a significant inverse correlation with the percentage of body fat in women and with glycemia in men (Figure 1). Linear regression analysis showed that these associations remained independent even after adjustments for age and SBP (Table 6).

**Table 5:** Pearson's correlation (r) of adiposity indexes and cardiovascular risk with fat percentage by sex

Variables	Women (n=48)		Men (n=33)	
	r	p value	r	p value
Body mass index (kg/m²)	0.556	< 0.001	0.738	< 0.001
Waist circumference (cm)	0.476	0.001	0.767	< 0.001
Waist-to-hip ratio	0.215	0.152	0.505	0.003
Waist-to-height ratio	0.550	< 0.001	0.767	< 0.001
Body adiposity index	0.599	< 0.001	0.653	< 0.001
Conicity index	0.264	0.076	0.597	< 0.001
Visceral adiposity index	−0.037	0.809	0.410	0.018
Vascular age (years)	0.062	0.682	−0.005	0.976
Cardiometabolic age (years)	0.242	0.109	−0.044	0.810
Metabolic syndrome criteria	−0.066	0.662	0.464	0.007

**Figure 1:** Correlation of the change in the area under the curve of skin perfusion by the laser speckle contrast imaging method with body fat in women and with glycemia in

**Table 6:** Linear regression of the change in the area under the curve (dependent variable) of skin perfusion by the laser speckle contrast imaging method with body fat in females and with plasma glucose in males after adjustment for age and systolic blood pressure

Dependent variables		Independent variables	Non-standard coefficient B	CI 95%	Standardized coefficient Beta	p value
AUC (%)	♀	Body fat (%)	−3.15	−6.29-0.10	−0.32	0.049
	♂	Glycemia (mg/dl)	−1.35	−2.47-0.22	−0.43	0.020

AUC: area under the curve;

♂ male;

♀ female.

## Discussion

This study evaluated the relationship between body adiposity and microvascular reactivity and their associations with different anthropometric and metabolic indexes in a population of treated hypertensive patients. No differences were found between the groups in peripheral and central pressure parameters, showing that the groups were hemodynamically well balanced.

Vascular and cardiometabolic ages were higher in the upper tercile fat percentage group, showing a positive association between body fat accumulation and vascular and metabolic damage. Considering that there was no significant difference in estimated cardiovascular risk, this finding reinforces the importance of assessing these parameters.

Higher values of uric acid and TG/HDL ratio were found in the tercile of higher fat percentage compared with the lower terciles. Elevated uric acid has been associated with metabolic syndrome. Experimental studies have suggested that uric acid can penetrate the smooth muscle and vascular fibers, culminating in increased expression of inflammatory mediators. The consequences are raised blood pressure and vascular smooth muscle cell hypertrophy.^17^^–^^19^ A recently published study conducted with adults in India demonstrated an association of uric acid levels with anthropometric obesity parameters such as BMI, WHR and WHtR.^18^

The TG/HDL ratio has been proposed as a simple marker of insulin resistance, acting as a biomarker to identify cardiometabolic risk profiles.^20^ Pantoja-Torres et al.^21^ demonstrated a positive association of TG/HDL ratio with insulin resistance in a eutrophic adult population. This association was also studied by Baez-Duarte et al.^22^ who found an association between the TG/HDL ratio with lower insulin sensitivity and the presence of metabolic syndrome in an adult population with an average BMI of 27.8 kg/m².

Since endothelial dysfunction is considered a marker of the atherosclerotic process, it is crucial to evaluate its earliest manifestations in micro and macrocirculation.^23^ The evaluation of endothelial function by microvascular reactivity through the LSCI method has not been used in clinical trials with an obese population.

In this study, microvascular reactivity was negatively associated with the accumulation of adipose tissue. Suboc et al.^24^ demonstrated that obesity was associated with worse endothelial function in non-diabetic adults. These findings only became significant when the groups were divided by gender.^24^ Endothelial function, assessed in the present study by the variation in AUC, correlated with plasma glucose in males, suggesting a possible important association between endothelial function and insulin sensitivity in men. In women, this correlation was more evident with body fat percentage, indicating a probable direct relationship between adipose tissue and vascular function in females. These correlations remained significant after adjusting for age and SBP, important factors in the regulation of endothelial function.

Regarding arterial stiffness parameters, no differences were found between the groups in the present study. Desamericq et al.^25^ did not find an association between obesity and increased arterial stiffness in adult subjects with associated CVR factors, such as diabetes mellitus. Menezes et al.^26^ did not find any association between obesity and insulin resistance with vascular alteration, in either endothelial function or arterial stiffness. In this study, the association of adiposity with arterial stiffness may have been attenuated by the effects of vasoprotective drugs, such as renin angiotensin system inhibitors (RASI).

The fat percentage obtained by BIA in males showed a good correlation with all anthropometric indices of body adiposity assessment, as well as the number of criteria for metabolic syndrome. In women, this correlation remained only for BMI, WC, WHtR and BAI. In 2007, a study conducted in Brazil, aimed at determining the association between the various obesity and coronary risk indicators, showed that the indicators were strongly associated with WHtR, with emphasis on WHR and CI among men, while CI was the best marker for women aged 50 to 74. This difference can be explained by the menopause, in which the loss of protection provided by estrogen occurs, leading to a higher accumulation of abdominal fat, which contributes to cardiovascular complications.^27^

Some limitations were identified in this study. The absence of inflammatory markers and adipokines impaired the analysis of inflammation as a mechanism of endothelial dysfunction associated with increased body adiposity. CRP was the only inflammatory marker evaluated and was not different among the study groups. The effects of some antihypertensive drugs may have influenced this result. No imaging method was used to quantify visceral adipose tissue. However, the main objective of the study was to use simpler methods to assess body adiposity and their association with vascular changes that may characterize a higher cardiovascular risk.

## Conclusion

In conclusion, in this sample of treated hypertensive patients, anthropometric obesity indexes were more associated with body fat percentage among men. The highest cardiovascular risk among those with higher body adiposity was more evidenced by the higher vascular and cardiometabolic ages in this group of patients. Higher body adiposity was associated with lower microvascular reactivity, which was more evident among women. There was no difference in arterial stiffness, which can be attributed to the use of antihypertensive medications that maintained similar blood pressure levels in the study groups.
